# Comparison of Mir122 expression in children with biliary atresia and healthy group

**DOI:** 10.22099/mbrc.2024.49649.1950

**Published:** 2024

**Authors:** Nasrin Motazedian, Negar Azarpira, Kimia Falamarzi, Seyed Mohsen Dehghani, Maryam Ataollahi, Elaheh Esfandiari, Mahintaj Dara, Razieh Toobafard, Mehrab Sayadi, Seyed Ali Shekarforoush, Seyed Hossein Owji, Seyed Ali Malekhosseini

**Affiliations:** 1 Transplant Research Center, Shiraz University of Medical Sciences, Shiraz, Iran; 2 Student Research Committee, Shiraz University of Medical Sciences, Shiraz, Iran; 3 Stem Cells Technology Research Center, Shiraz University of Medical Sciences, Shiraz, Iran; 4 Cardiovascular Research Centre, Shiraz University of Medical Sciences, Shiraz, Iran; 5 Otolaryngology Research Center, Department of Otolaryngology, Shiraz University of Medical Sciences, Shiraz, Iran; 6 Abu-Ali Sina Organ Transplant Center, Shiraz University of Medical Sciences, Shiraz, Iran

**Keywords:** Micro RNA, miR-122, Biliary atresia, Liver

## Abstract

Biliary atresia (BA) is the primary cause of neonatal jaundice with various pathological mechanisms. Many BA patients may experience progressive liver dysfunction and eventually need a liver transplant. Therefore, identifying potential non-invasive biomarkers for BA is crucial. miR-122, the most abundant microRNA in the liver, plays significant roles in different liver diseases. This study aimed to assess miR-122 levels in BA patients. Eighteen patients with biliary atresia were selected at random from the Shiraz Pediatric Liver Cirrhosis Cohort Study (SPLCCS), along with 18 healthy controls. Blood samples were collected, and biochemical parameters (such as liver function tests) were measured. Quantitative reverse-transcription PCR (RT-PCR) was conducted on serum samples from both the case and control groups to analyze miR-122 levels. The study results indicated that serum miR-122 expression in BA patients was elevated compared to the control group, although it did not reach statistical significance. Additionally, no correlation was found between miR-122 expression and serum levels of liver enzymes or other laboratory findings in BA cases. miR-122 could be a potential target for diagnosing BA; however, further research with a larger population is necessary to determine if miR-122 could serve as a useful biomarker for diagnosing BA.

## Introduction

Biliary atresia (BA) is a progressive fibroinflammatory cholangiopathy that is the primary reason for neonatal jaundice and has multiple contributing factors [1, 2]. Viral infections, environmental toxins, genetic predispositions, autoimmune responses, inflammation, morphology, and vascular defects are among the pathomechanisms of BA which involve both intrahepatic and extrahepatic biliary tree [3]. Persistent jaundice, conjugated hyperbilirubinemia, clay-colored stool, and dark urine are among the clinical presentations of BA, and if untreated, it leads to hepatic fibrosis, portal hypertension, and end-stage liver disease [4-6]. Laboratory findings, and different radiologic methods such as ultrasound, hepatobiliary technetium-labelled iminodiacetic acid (HIDA) scan, magnetic resonance cholangiopancreatography (MRCP), and endoscopic retrograde cholangiopancreatography (ERCP) have been reported as diagnostic methods for BA [4, 5]. Accurate diagnosis of BA and the stage of fibrosis can be established by liver biopsy, which is an invasive technique [1]. Kasai portoenterostomy (KPE) is the current treatment option for BA patients, and its success rate is mostly based on patients’ age; however, despite improvements in BA outcome due to KPE, biliary cirrhosis, progressive fibrosis, and liver dysfunction may occur in most patients [1, 3, 7]. Consequently, liver transplantation is required for post-KPE failures, and it is still the ultimate measure for treating more than 70% of BA patients [6, 8]. MicroRNAs are small (18–23 nucleotides in length), non-coding RNAs that are implicated in regulating gene expression by modulating mRNAs post-transcriptionally, and abnormal miRNA expression might result in the development of various diseases. Circulating microRNAs may be proposed as potential biomarkers for predicting several pathologies [7, 9, 10].

miR-122 is a liver-specific miRNA that constitutes 70% of all hepatic miRNAs and plays a crucial role in hepatocyte proliferation, differentiation, and apoptosis [11]. Prior research has suggested that miR-122 facilitates the replication of hepatitis C virus (HCV) and could be a promising target for HCV therapy. [12]. Conversely, miR-122 inhibits hepatitis B virus (HBV) expression and replication through an enhancer regulatory pathway. This approach elevates miR-122 levels as an effective strategy to restrict HBV infection or impede the progression of hepatocellular carcinoma (HCC) in the presence of HBV infection [13]. Additionally, miR-122 is significantly involved in lipid metabolism and may safeguard the liver against lipid metabolic disorders like NAFLD; as inhibition of miR-122 reduces lipogenesis by suppressing lipogenic genes. Hence, miR-122 holds promise as both a biomarker and treatment for NAFLD in the future [14]. Furthermore, miR-122 could serve as a potential biomarker in various liver conditions such as NAFLD, NASH, liver injury caused by drugs, drug-induced cholestatic liver disease, hepatic fibrosis, and cirrhosis [14-16]. 

The Shiraz Organ Transplant Center is the primary facility for pediatric liver transplants in Iran, where cirrhotic children requiring liver transplantation from all regions of Iran are referred. Consequently, the Shiraz Transplant Research Center has initiated the Shiraz Pediatric Liver Cirrhosis Cohort Study (SPLCCS) [17]. Data and blood samples from children with biliary atresia (BA) enrolled in the SPLCCS were accessible. Therefore, we conducted a study to identify noninvasive biomarkers that could facilitate early diagnosis of BA patients.

## MATERIAL AND METHODS

**This cross-sectional study was based on SPLCCS with a control group: **The study involved 18 children diagnosed with BA, and informed consent was obtained before their enrollment in SPLCCS. Blood samples collected and preserved in the biobank at the Shiraz Transplant Research Center. [17]. Data from laboratory tests and clinical assessments of the children were retrieved from the pediatric liver cirrhosis registry (IR.SUMS.REC.1399.530). The control group comprised 18 healthy children from the surgery wards scheduled for minor procedures like tonsillectomy. Informed consent was obtained from their parents before sampling (IR.SUMS.MED.REC.1401.466).

**Circulating microRNAs and quantitative real-time polymerase chain reaction: **miRNA was isolated from serum samples of BA patients using RNX-Plus (Cinnagen, Iran) as per the manufacturer's guidelines. Subsequently, complementary DNA was generated employing the RB-Micro RNA Synthesis kit (RNA biotechnology company, Iran) with specific stem-loop primers and 1000 ng total RNA in a 20-μL reaction. The reaction comprised 100 pMol stem-loop primer for MicroRNA, 10 pMol RT primer, 10 mM dNTP, 5× Reverse Transcriptase Buffer, and M-MLV reverse transcriptase enzyme, conducted in a thermal cycler machine [18] under the following conditions: 12 minutes at 65°C, 55°C for 50 minutes, 72°C for 20 minutes, and 4°C for 10 minutes. Real-time PCR was carried out using Step-One ABI Applied Biosystems (Life Technologies). U6 snRNA was used as a control for miRNA-122 expression analysis. The PCR reactions were performed with the following parameters: 95°C for 15 minutes, 95°C for 20 seconds, and 62°C for 60 seconds for 40 cycles. Each sample was tested three times, and the average cyclic threshold (Ct) values were utilized for analysis. The Livak (2^−ΔΔCt^) method was employed to assess miRNA-122 expression in the samples.

**Statistical Analysis: **The data were reported as mean±SD or number (%) for continuous and categorical variables, respectively. Data normality was evaluated using the Shapiro-Wilk test, skewness, and kurtosis indices. Statistical comparisons between the two groups were performed using the Student’s t-test (or Mann-Whitney test) for continuous variables and the Chi-square test for categorical variables. Additionally, the relationship between miR-122 expression and laboratory findings, baseline characteristics, and gender in the cases was analyzed using Spearman’s test and Mann-Whitney test. Data analysis was carried out using SPSS software (Version 16, SPSS Inc., Chicago, United States). A P-value<0.05 was considered statistically significant.

## Results

The study included a total of 18 patients with BA and 18 control subjects, with their demographic and clinical profiles detailed in Tables 1 and 2. In the BA group, nine out of 18 patients (50%) were female, with a mean age of 2.91±3.95 years, while the control group had a mean age of 5.98±2.37 years, with 50% being female. Liver enzyme levels (such as AST, ALT, ALKP) were notably higher in the serum of BA patients compared to the healthy participants. Among the BA patients, 11.1% had a family history of liver diseases, and 38.9% had relatives who were parents. Liver transplantation was undergone by 38.9% of BA patients, and 83.3% had Kasai portoenterostomy (KPE). 10 patients (55.6%) with BA passed away.

**Table 1:** Demographic and clinical findings of the biliary atresia group and control groups

**Variables**	**Biliary atresia group** ** (** **N=18)**	**Control group (N=18)**	**P-value**
Age (years)	2.91 ± 3.95 (0.2-12.8(	5.98 ± 2.37 (3.0-12.0(	0.008
Height (Cm)	81.1 ± 30.4	-	
Weight (Kg)	12.2 ± 10.6	-	
Gender Male Female	9 (50) 9 (50)	9 (50) 9 (50)	>0.999
Parents are relative First degree relative Second degree relative	7 (38.9) 4 (57.1) 3(42.9)	-	
Liver disease in family	2 (11.1)	-	
Liver transplantation	7 (38.9)	-	
Surgery Kasai procedure	15 (83.3)	-	
Death	10(55.6)	-	

Data were presented as mean ± SD (range) for continues and number (percent) for categorical variables.

**Figure 1:**
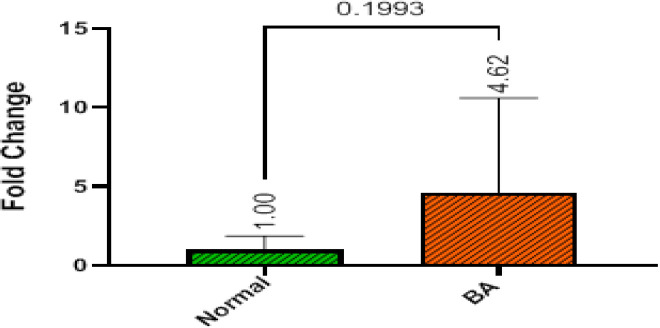
miR-122 expression levels in the BA patients and control group

The study assessed miR-122 expression levels in the serum of both BA patients and healthy control subjects. Results indicated that miR-122 expression was higher in the serum of BA patients (mean fold change of 4.62) compared to the control group (mean fold change of 1.00); however, this difference did not reach statistical significance (p=0.1993) (Fig. 1).

**Table 2:** Laboratory findings of the biliary atresia group and control groups

**Variables**	**Biliary atresia group** **)N=18)**	**Control group (N=18)**	**P-value**
**AST (U/L)**	284 ± 625.25 )21–2712(	23 ±7.83 )1-38(	<0.001
**ALT (U/L)**	182.8 ± 301 )10-1250(	8.33 ± 3.98 )2-15(	<0.001
**ALK.P (U/L)**	837.27 ± 651.40 (201-2971)	430.16 ± 136.06 (319–898)	<0.001
**Albumin (g/dL)**	3.18 ±0 .75 (0.7-4.30)	3.88 ± 0.42 (3-5)	<0.001
**Total Protein (g/dL)**	5.56 ± 1.35 (2.10-7.50)	5.85 ± .81 (4.7-7.7)	0.789
**Total Bili (mg/dL)**	7.04 ± 7.23 (0.3-24.1)	0.46 ± .11 (0.4-0.8)	<0.001
**Direct Bili (mg/dL)**	5.42 ± 8.65 (0.05-35.84)	0.20 ± 0.11 (0.1-0.05)	<0.001
**Hb (g/dL)**	9.58 ± 1.29 (7.6-12.3)	12.8 ± 1.21 (10.3-14.5)	<0.001
**WBCs**	11016 ± 6405 (1400-22300)	7970 ± 1937 (5100–11500)	0.207
**PLT**	232555 ± 159979 (22000–572000)	332454 ± 53068 (255000-428000)	0.024
**INR**	4.25 ± 10.46 (1-44.2)	-	
**PT**	19.31 ± 18.86 (13-92)	-	
**GGT (U/L)**	313 ± 329.78 (11–900)	-	

Data were presented as mean ± SD (range)

The investigation explored the correlation between miR-122 expression and baseline characteristics and laboratory values in BA patients. No significant correlation was observed between serum miR-122 levels and laboratory parameters such as ALT, AST, ALKP, and GGT in BA patients (p>0.05), as detailed in Table 3. Similarly, there was no statistically significant link between serum miR-122 levels and baseline demographic factors including gender, parental relation, KPE, liver transplantation, and mortality (p>0.05, Table 4).

**Table 3:** Correlation between miR-122 expression levels and laboratory data

**Variables**	**p-value ** ^a^	**rho coefficient**
**AST**	0.624	-0.124
**ALT**	0.711	-0.94
**ALKP**	0.576	-0.141
**Alb**	0.974	0.009
**Total protein**	0.562	0.163
**Total Bilirubin**	0.498	-0.176
**Direct Bilirubin**	0.613	-0.132
**Hb**	0.315	0.251
**WBC**	0.515	0.164
**Platelet**	0.610	-0.129
**INR**	0.237	-0.303
**PT**	0.397	-0.220
**GGT**	0.623	0.257

Data were presented as rho (correlation coefficient ), ^a ^P value extract form Spearman’s test

**Table 4:** Comparison of miR -22 according to different categories of study variables

**Variables**	**Median (IQR)**	**P value** ^a^
**Age**		0.964
<3	1.70 (0.32-7.05)	
4>=	1.70 (0.61–9.60)	
**Gender**		0.730
Male	0.88 (0.48–6.32)	
Female	5.75 (0.40-8.45)	
**Liver transplantation**		>0.999
Yes	1.84 (0.23 -9.45)	
No	1.56 (0.59 -7.46)	
**Surgery**		0.498
Yes	1.56 (0.23 – 9.45)	
No	5.83 (0.88 -5.83 )	
**Parents relation**		0.536
Yes	1.56 (0.84 – 9.45)	
No	1.84 (0.22 -7.46)	
**Death**		0.633
Yes	1.22 (0.50 – 6.24)	
No	0.35 (2.20 -11.43)	

Data were presented as median (IQR) , ^a^Man-Whitney U was used

## Discussion

Today, the use of miRNAs as therapeutic targets or non-invasive methods in the diagnosis of diseases such as infectious diseases, genetic diseases, and various cancers is highly regarded [19]. In general, changes in miRNA expression are linked to liver conditions such as disrupted liver metabolism, liver injury, liver fibrosis, and tumor development, rendering miRNAs attractive therapeutic candidates for the detection and management of liver ailments. Biliary atresia (BA) is a common liver disorder in children, emphasizing the importance of early BA diagnosis to improve outcomes post-Kasai portoenterostomy (KPE) [1, 20].

This study aimed to investigate serum miR-122 expression levels in patients with biliary atresia (BA) and assess its potential as a diagnostic or therapeutic target for pediatric liver conditions. While the expression of miR-122 in the serum of BA patients was elevated, the difference compared to the control group did not reach statistical significance. Zahm et al. found no significant differences in miR-122 serum levels between BA patients and cholestatic controls, indicating that hepatocytes may have undergone similar injury in both groups. However, the expression of the miR-200b/429 cluster was notably higher in the sera of BA patients compared to cholestatic controls. [21].

Murray et al. identified miR-122 as a promising novel biomarker for liver disease in HIV-infected individuals undergoing anti-retroviral therapy [22]. Rachel et al. studied the miRNA profile during hepatobiliary injury over time. They found that miR-122 significantly increased during hepatic injury from toxic substances and returned to baseline levels during the repair phases, suggesting its potential as a biomarker for hepatotoxicity [23]. Calkins et al. demonstrated the association of serum miR-122 with liver disease in children with intestinal failure [24]. Peng et al. also highlighted plasma miR140-3p as a diagnostic marker for biliary atresia using NGS [25].

Smith et al. delved into the causes of biliary atresia (BA) and identified several potential biomarkers linked to liver and bile duct development, as well as liver inflammation. They observed that miR-30 and the miR-23 cluster play a role in liver and bile duct development, while miR-29, miR-483, miR-181, miR-199, and miR-200 are involved in inflammation and fibrosis [26]. The obstruction of the extrahepatic biliary tract in BA leads to damage in hepatocytes or cholangiocytes. Therefore, an elevation in serum miR-122 serves as a general indicator of liver injury rather than a specific disease marker [27]. Evidence suggests that miR-122 serves as a biomarker for various liver diseases in both adults and children [21, 24, 28]. However, limited research has explored the expression of miR-122 in children with BA, and the findings from these studies are conflicting and complex [25, 29, 30]. The discrepancies among studies may stem from differences in the control group characteristics. Comparisons with a healthy control group might have revealed distinct miR-122 expression patterns, but when compared to other cholestatic diseases, the overexpression of miR-122 was not observed.

The results of our study did not find any correlation between liver enzymes (AST, ALT, ALKP, GGT), serum bilirubin, total protein, and albumin levels and miR-122 expression. Another investigation also found no relationship between miR-122 serum levels and albumin, total protein, and bilirubin levels in cirrhotic patients [31]. However, previous studies have shown a positive association between miR-122 and ALT and AST serum levels, indicating an earlier and greater increase in miR-122 levels compared to aminotransferases levels in patients with liver injury [18, 32-34]. Pirola et al. also demonstrated a positive correlation between miR-122 and GGT levels in NAFLD patients. They further suggested that miR-122 might upregulate ALT protein translation, a marker for liver injury, leading to increased enzyme activity [35]. Although no link was found between miR-122 expression levels and CBC, PT, and INR in BA cases, Waidmann and colleagues identified a negative correlation between miR-122 and INR in patients with liver cirrhosis [31]. These discrepancies in results could be attributed to the limited sample size in our study or variations in the pathogenesis of liver diseases. Our findings indicated no relationship between miR-122 levels and parents' relation, KPE, or liver transplantation among BA patients. In this study, the available blood samples of BA patients in the cohort bank were utilized. This study represents the first investigation into the role of miR-122 as a diagnostic marker in BA children in Iran.

In conclusion, the findings of the present study revealed a rise in miR-122 serum levels in BA cases compared to healthy controls; nevertheless, it was not statistically significant. miR-122 could serve as a promising non-invasive diagnostic and prognostic biomarker for BA patients. Hence, further research is warranted to explore and evaluate suggested miRNAs as diagnostic and prognostic markers in a larger sample size for early detection and improved patient outcomes.

## Conflict of Interest:

The authors declare that there is no conflict of interest.

## Authors’ Contribution:

All of the authors contributed substantially to the concept and design of the study. Material preparation, data collection, and analysis were performed by NM, NA, KF, SMD, MA, ES, MD, MS, S.Sh, SHO, and SAM. The primary draft of the manuscript was written by NM. All authors read and approved the final manuscript.
